# The effects of daily step goals of 10,000, 12,500, and 15,000 steps per day on neural activity to food cues: A 24‐week dose‐response randomized trial

**DOI:** 10.1002/brb3.2590

**Published:** 2022-04-16

**Authors:** Sharla E. Compton, Michael J. Larson, James D. LeCheminant, Larry A. Tucker, Bruce W. Bailey

**Affiliations:** ^1^ Department of Exercise Sciences Brigham Young University Provo Utah USA; ^2^ Department of Psychology Brigham Young University Provo Utah USA

**Keywords:** appetite, event‐related potential, late positive potential, physical activity, young adult

## Abstract

**Introduction:**

The purpose of this study was to examine the effect of different levels of sustained physical activity on neural reflections of attention allocated toward food cues in first year college women.

**Methods:**

Seventy‐nine first‐year college women (18.6 ± 0.5 years) were recruited to participate in the study. Women were randomly assigned to a daily step goal of 10,000, 12,500, or 15,000 for 24 weeks. Once during weeks 16–24, participants were shown pictures of plated meals or flowers with the neural response measured using the P300 and late positive potential (LPP) components of the scalp‐recorded event‐related potential. Diet was assessed using the automated 24‐h recall.

**Results:**

Both the P300 and LPP amplitudes were significantly more positive to food versus flower pictures (*p*s < .001). There was no interaction between step group and picture condition for the P300 and LPP. However, the 12,500‐step group showed a significantly elevated LPP amplitude in comparison to the other groups for both food and flowers (*F*(2,74) = 8.84; *p* < .001). The effect size for the combined results (food and flowers) was 0.56 between 10,000 and 12,500‐step groups, and 0.75 between the 12,500‐ and 15,000‐step groups. In addition, the 12,500 group reduced caloric consumption over the course of the intervention (*t*(1,74) = 3.35, *p* = .001, *d_z_* = 0.59).

**Conclusion:**

Habitual physical activity of 10,000, 12,500, or 15,000 steps per day does not preferentially alter neural reflections toward food cues compared to flowers. There may be a nonlinear response to pleasant visual cues, with 12,500 steps per day eliciting higher LPPs than either 10,000 or 15,000.

## Graphical abstract

The purpose of this study was to examine the effect of different levels of sustained physical activity on neural reflections of attention allocated toward food cues in first year college women. Habitual physical activity of 10,000, 12,500, or 15,000 steps per day does not preferentially alter neural reflections toward food cues compared to flowers. There may be a nonlinear response to pleasant visual cues, with 12,500 steps per day eliciting higher LPPs than either 10,000 or 15,000.

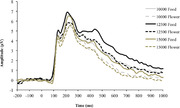

## 1 INTRODUCTION

Risk of weight gain is elevated during the first year of college (Hajhosseini et al., 2006). College freshmen tend to gain weight at a rate that is higher than the general population (Butler et al., 2004; Finlayson et al., 2012; Hajhosseini et al., 2006; Smith‐Jackson & Reel, 2012). This increased rate of weight gain is likely a result of the many environmental and social changes experienced during this transitional period of life (Childers et al., 2011; Filla et al., 2013; Racette et al., 2005).

One health‐related behavior that may help explain the weight gain seen in college freshmen is decreased physical activity (Bailey et al., 2015, 2014; Jung et al., 2008). A significant decline in physical activity among 18‐ to 29‐year olds has been found in several studies (Butler et al., 2004; Filla et al., 2013; Jung et al., 2008; Leslie et al., 2001; Pullman et al., 2009; Racette et al., 2005). Additionally, a decrease in physical activity has been observed in individuals during the transition from high school to college (Filla et al., 2013; Pullman et al., 2009). University freshmen report significantly more barriers to physical activity than do grade school or high school students (Gyurcsik et al., 2006), and 30% of first‐year college students reported that they do not exercise (Racette et al., 2005).

One method of measuring habitual physical activity is by pedometry. Pedometers are a reliable and effective method to measure free‐living physical activity in college students (Bailey et al., 2014; Felton et al., 2006; Jackson & Howton, 2008). Pedometers are also an effective method of influencing habitual physical activity, and there is now an extensive research base that supports the use of pedometers for increasing physical activity (Bailey et al., 2019; Jackson & Howton, 2008; LeCheminant et al., 2011; Morton et al., 2015; Over et al., 2012).

Although physical activity is an important part of weight management, the influence of physical activity on weight loss is often less than expected (Donnelly et al., 2009; Laddu et al., 2011; Tate et al., 2007; Wing & Phelan, 2005). This is because energy intake also plays an important role in energy balance and, consequently, body weight. Physical activity and energy intake are not independent, and increased energy expenditure may alter attention allocation toward food (Hanlon et al., 2012). This alteration could make resistance to food cues more difficult, thus altering energy consumption and attenuating the impact of physical activity on body weight.

Although studies that have evaluated subjective feelings of hunger and appetite suggest an interaction between exercise and attention allocation toward food, objective measures may be more sensitive to change and better describe this relationship. One method used to measure the time course of neural electrical activity associated with viewing food stimuli is through scalp‐recorded event‐related potentials (ERPs). ERPs represent the averaged electrical activity of the brain time‐locked to the presentation of stimuli (Hajcak et al., 2010).

Two common ERP components that are used to assess attentional processing of external cues is the P300 and late positive potential (LPP). P300 is the nomenclature describing a positive deflection in an ERP peaking around 300 ms following the stimulus and suggests initial increased attention to a stimulus (the P300 is also referred to as the third positive peak in the ERP after stimulus presentation). P300 is one of the most studied ERP components (Patel & Azzam, 2005). Large‐scale reviews on the functional significance of the P300 suggest that it represents contextual updating processes following stimulus presentation or the relatively rapid representation of attention resource allocation to motivationally‐relevant stimuli (Polich, 2007; Schupp et al., 2007). P300 amplitude is increased when individuals find stimuli emotionally arousing/intense and is larger to emotional picture stimuli compared to neutral (Yuan et al., 2019). From a food stimulus perspective, P300 tends to be increased to high‐calorie food pictures compared to neutral items or low‐calorie food pictures, though this pattern is not consistent in all studies (Allen et al., 2022; Carbine et al., 2020, 2018; Chami et al., 2019).

The LPP is a slower potential that begins between 300 and 700 ms after the presentation of the stimulus (Cuthbert et al., 2000) and is functionally associated with increased attentional processing of emotional or motivational images and cues (Hajcak et al., 2010). Some suggest that the LPP is distinctly related to attentional‐bias toward pleasing or appetitive stimuli (Gable & Harmon‐Jones, 2010). Hajcak and Olvet (2008) show that the LPP is larger after emotional images than neutral images and may represent attentional capture and processing of emotional information well after stimulus presentation. The LPP is also related to emotional reactivity and emotional regulation (Hajcak et al., 2014), with a growing consensus that the LPP represents an automatic sustained engagement to content with strong emotional significance (Hajcak & Foti, 2020). In the context of food‐related picture viewing, LPP amplitude has a larger sustained response to food stimuli relative to neutral and, similar to P3 amplitude, to high‐calorie stimuli relative to low‐calorie stimuli (Carbine et al., 2021, 2018; Chami et al., 2019; Sarlo et al., 2013).

Objective measures of neural activity have been used to examine changes in attention allocation toward food following exercise (Cornier et al., 2012; Evero et al., 2012; Hanlon et al., 2012). For example, Evero et al. (2012) found in a short‐term crossover study that functional magnetic resonance imaging (fMRI) scans showed reduced neural response to acute exercise in brain regions involved in food reward and visual attention. Hanlon et al. (2012) similarly tested the response of exercise on neural reflections to food cues using electroencephalogram (EEG). The results confirmed that of Evero et. al (2012); namely, following an acute bout of exercise, a decreased neural response to food cues was observed relative to when the participants did not exercise. More specifically, LPP amplitude was decreased to food pictures following exercise, although the amplitude of the LPP was not related to self‐reported food intake. Other studies that have tested food‐related attention allocation suggest increased LPP amplitude to food‐related stimuli relative to other positive/hedonic images (e.g., flowers) (Feig et al., 2017; Stockburger et al., 2009).

Neural responses to food cues can change following acute and prolonged exercise. For example, in a large‐scale study of over 200 participants using a within‐subjects crossover design, Bailey et al. (2021) showed that N2 and P3 ERP amplitudes were larger when inhibiting high‐calorie food stimuli following high‐intensity acute exercise (70% VO_2 Max_) compared to lower intensity exercise (30% VO_2 Max_) or rest. Cornier et al. (2012) used visual analog scales (VAS) and fMRI before and after a progressive 6‐month exercise program on 12 overweight or obese participants (five women, seven men) (Cornier et al., 2012). Neural reflections of the response to food were found to decrease in the posterior attention network and insula, while there were no changes in hunger, satiety, or prospective food consumption. However, it is difficult to come to any firm conclusion about these relationships given the limited sample size, lack of a comparison group and sample randomization.

Several studies have looked at step levels in college women and how daily steps influence body weight and adiposity. LeCheminant et al. (2011) observed that randomly assigning participants to 10,000 steps per day versus normal activity levels during two semesters of college resulted in similar weight gain. Unfortunately, this study did not assess baseline steps and failed to report the average steps per day in the control group (no step recommendation). However, the authors did speculate that it is likely that control condition already was close to accumulating 10,000 steps per day because of the nature of campus life. Additionally, Bailey et al. (2014) observed that daily steps were related to body weight and adiposity up until 11,000 steps per day. In this observational study, going beyond 11,000 steps per day added no additional benefit for weight and adiposity (Bailey et al., 2014). The results of these studies suggest that compensation may take place as physical activity progressively increases beyond 10,000 steps per day. One area of compensation may be related to food. As physical activity levels increase it is possible that attention allocation to food cues also increases to maintain energy balance.

To our knowledge, a study evaluating a variety of prolonged physical activity levels (sustained for at least 16 weeks) using ERPs to measure neural reflections of processing of food cues with an adequate sample has not been done. The purpose of this study was to examine the effects of progressively higher levels of prolonged physical activity (10,000, 12,500, and 15,000 steps per day) on ERP indices of attention to pictures of food in women during the first year of college. We chose 2500 step increments because this represents about a mile of walking between conditions (Hoeger et al., 2008). We hypothesized that there would be a dose‐response relationship observed with women who habitually accumulate the highest steps counts, expressing higher attention allocation toward food (i.e., increased amplitude of the LPP and P300 to food cues relative to control cues).

## 2 MATERIALS AND METHODS

### 2.1 Research design

All study procedures were approved by the University Institutional Review Board, and participants provided written informed consent. The study took place at one university in the mountain west part of the United States. All participants were university students, and the study took place in this university setting. We used a randomized three‐group posttest experimental design. We selected this design since entry to the study was rolling during the initial part of the school year and it was not feasible to perform the EEG pretest and still have time for all the participants to complete the study by the end of the academic year. (Marczyk et al., 2005) The posttest experimental design controls for the same threats to validity as a randomized pre‐ and posttest design and has the added benefit of controlling for or eliminating the threat to internal validity of pretesting and instrumentation (Marczyk et al., 2005). No changes were made to the study design or methods once the study began.

We used a computer‐based random number generator to assign participants to groups. Randomization was performed by the senior author (B.B.). Randomization took place after baseline assessment. No effort was taken to blind the researchers or participants from the intervention assignment.

Participants were randomly assigned to accumulate 10,000, 12,500, or 15,000 steps per day. These recommendations either meet or exceed current step recommendations for freshman‐aged individuals and represent a progressive 25% increase in physical activity from one recommendation to the next (Tudor‐Locke et al., 2011). We wanted all groups to be physically active, while separating progressive recommended levels by a large enough margin to evaluate health differences due to activity level. The 2500 step difference between the three progressive groups required approximately an extra mile per day, and this level of separation in steps has been previously used in intervention studies (Tudor‐Locke et al., 2011). Participants were instructed to accumulate their step recommendation daily for 24 weeks. Between weeks 16 and 24, participants came to the lab for the EEG data collection session.

### 2.2 Participants

Participants were studied in two cohorts corresponding to the beginning of the fall and spring semesters during the university school year. All participants were freshmen women during their first term or semester at college at the time of recruitment. Recruitment was through flyers, a booth at the new student orientation fair, and word of mouth. Participants were required to be able to participate in physical activity without restriction, not be taking metabolic‐altering medications of any kind, and have a body mass index (BMI) ≥ 20 (kg/m^2^). To ensure accurate ERP data, participants were excluded if they had a history of a head injury resulting in unconsciousness or a neurological disorder such as epilepsy or stroke. Three participants were excluded from the analysis for history of head injury (10,000 *n *= 1; 12,500 *n* = 1; 15,000 *n* = 1). Those participants who self‐reported exercising more than three times per week for more than 30 min per exercise session were not enrolled in the study. In addition, participants who averaged more than 10,500 steps per day during their baseline assessment were excluded from the study. We chose 10,500 steps because we did not want women who were already habitually meeting higher step counts to be randomized to a lower step count condition. We also recognized that students are likely to be a little more active at the beginning of the school year as they get used to their new schedules. Recruitment began in September 2013, and data collection for the study was completed by April 2015.

### 2.3 Procedures

Following inclusion screening, participants were given a pedometer to wear for 4 days (two weekdays and two weekend days) to evaluate baseline step counts. In addition, participants completed three 24‐h recalls. After baseline steps and diet were assessed, height and weight were measured and participants were randomized into one of three step groups: 10,000, 12,500, or 15,000 steps/day. Participants were instructed that they were expected to achieve their step recommendation at least 6 days of the week. Although not required to make their steps on the seventh day, participants were instructed to still wear the pedometer the entire day. Additionally, participants were instructed not to discuss their step group with other participants. Every other week, participants came to the research lab to download data from their pedometers. After data were downloaded, a research assistant and the participant reviewed the step data together. If the participant was having trouble meeting their step goal the research assistants address this specifically and worked with the participant to make plans to improve over the next 2 weeks. Participants were not dropped from the study if they were having difficulty meeting their step goals.

After at least 16 weeks of intervention, participants were scheduled on a weekday morning between 8:00 a.m. and 10:00 a.m. for an EEG/ERP recording session. Participants reported for the EEG session in an 8‐h fasted state to ensure similar levels of food intake between all three groups. Participants were also asked not to exercise in the morning prior to the EEG assessment. Following application of the EEG sensors, participants completed an experimental task focused on the passive viewing of pictures of food and flowers that we have used previously to study attention to food cues (Hanlon et al., 2012). Specifically, the task consisted of 240 total picture presentations (50% food, 50% flower) divided into three blocks of 80 pictures (40 pictures of food and 40 pictures of flowers) presented in random order. All pictures of food contained multiple foods per plate except one (a single picture of a spaghetti noodle‐based meal). Pictures included salads (six pictures), fruits (two pictures), meat‐based meals (15 pictures), sandwiches (five pictures), pizza/tacos/nachos (three pictures), dessert (three pictures), pasta‐based meals (five pictures), and pancakes (one picture). Due to similarity in color and appearance to pictures of plated food, flower pictures were chosen to control for increased attention toward food (Stockburger et al., 2009). Picture stimuli were matched for size, contrast, brightness, and intensity and were presented for 2000 ms followed by a 500 ms inter‐trial interval on a 17‐inch computer monitor approximately 20 inches from the participant. Following the EEG recording, participants rated the valence (i.e., pleasantness) of the picture on a nine‐point scale from extremely unpleasant to extremely pleasant and arousal (i.e., intensity) of the picture on a nine‐point scale from not at all arousing to extremely arousing or intense. Participates were compensated $150 for full participation and completion of the study.

### 2.4 Measurements

#### 2.4.1 Physical activity

Steps were recorded for each participant using an Omron pedometer (Omron Healthcare, Inc., Bannockburn, IL, USA). The Omron HJ720ITC multi‐function downloadable pedometer can store 41 days of data and is capable of distinguishing aerobic steps from total step counts while worn at multiple places on the body. Validity and reliability of this pedometer model have been previously documented (Holbrook et al., 2009). Placement position has also been evaluated (midback, backpack, right pocket, left pocket, right hip, and left hip). All placement positions were found to be valid and to give equivalent results, with the exception of the backpack position. For convenience, participants were instructed to wear the pedometer over their right hip or in pockets that restrict the pedometer from flipping over (e.g., such as jean pockets that would hold the pedometer to the body). For reliability in the current study, coefficients of variance were calculated. Under self‐paced walking conditions, the coefficients of variance were 1.4%. There was no significant difference in coefficients of variance across walking speeds using repeated‐measures analysis of variance (ANOVA) (Holbrook et al., 2009).

#### 2.4.2 Electrophysiological (EEG) data recording and analysis

We followed the methodology of our previous study on exercise and food‐related attention for the EEG recording and analysis (Hanlon et al., 2012). Specifically, EEG was recorded from 128 scalp sites using a geodesic sensor net and Electrical Geodesics, Inc. (EGI; Eugene, Oregon) amplifier system (20K nominal gain, bandpass = 0.10–100 Hz). Electroencephalogram was initially referenced to the vertex electrode and digitized continuously at 250 Hz with a 24‐bit analog‐to‐digital converter. Impedances were maintained below 50 kΩ. Data were average re‐referenced off‐line and digitally low‐pass filtered at 30 Hz.

Eye blinks were removed from the segmented waveforms using independent components analysis implemented in the ERP PCA Toolkit (Dien, 2010) (where PCA is principal component analysis). The independent components analysis for artifacts correlated at 0.9 with the scalp topography of two blink templates, one generated based on the current data and another provided by the ERP PCA toolkit author, were removed from the data (Dien, 2010). Trials were considered bad if more than 15% of channels were marked bad. Channels were marked bad if the fast average amplitude exceeded 100 μV or if the differential average amplitude exceeded 50 μV. Averaged ERPs were stimulus‐locked to the picture from 200 ms prior to picture presentation and 1000 ms poststimulus. We used the 200 ms prestimulus window as the baseline adjustment period.

**FIGURE 1:**
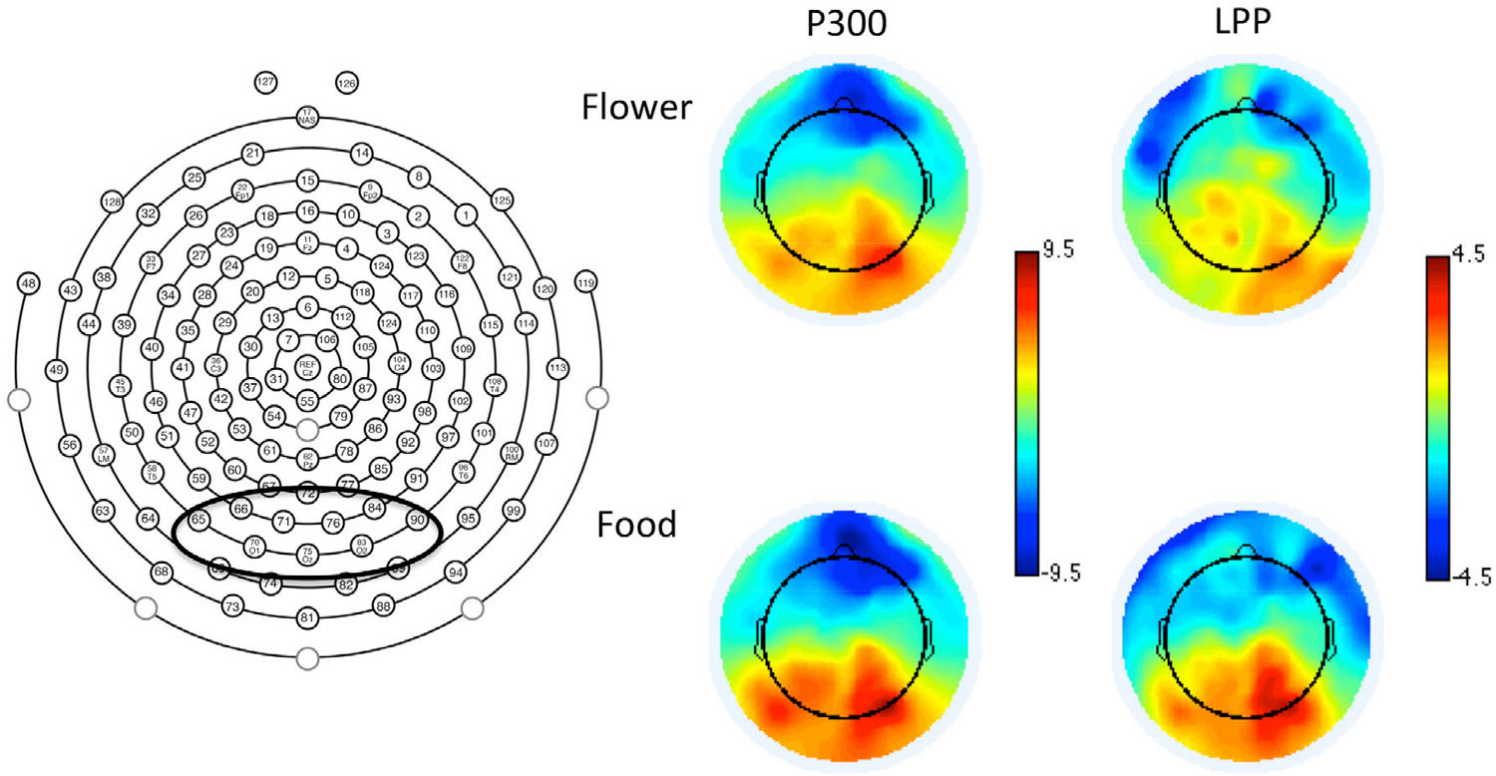
Left: EGI sensor net with the electrodes averaged together for analysis included in the dark circle. Right: Voltage maps for food and flower pictures collapsed across groups. Voltage maps for the P300 represent the average from 215 to 265 ms; for the late positive potential (LPP) from 600 to 700 ms

Previous research suggests increased ERP reliability when averaging across multiple sensors (Huffmeijer et al., 2014). Thus, we averaged across medial‐posterior sites just posterior to Pz, including sites 65, 66, 70, 75 (Oz), 76, 83, 84, and 90 (see Figure 1). Electrode sites and component timings were chosen using a collapsed localizer approach wherein the grand average including all conditions was examined for the largest peak for the P300 component and clearest LPP and scalp distribution (Luck & Gaspelin, 2017). Once the scalp distribution and component timings were chosen from the collapsed data, the data were separated and analyzed by condition. The scalp distribution and timings were generally similar, though not exact, with those used in our previous study using the same stimuli (Hanlon et al., 2012). The P300 was quantified as the mean amplitude from 215 to 265 ms. Latency of the P300 was determined using the maximal peak positive amplitude in the same window. The LPP was quantified as the mean amplitude from 600 to 700 ms (Moran et al., 2013). See Figure 3 for the P300 and LPP ERP waveforms. No latency calculations were made for the LPP as it is a slow tonic component without a clear peak.

All individual‐subject averages contained a minimum of 30 trials to ensure adequate signal‐to‐noise ratio. Previous studies show that as few as 8 or 12 trials are needed for reliable LPP amplitudes (Moran et al., 2013). A 3‐Group × 2‐Picture ANOVA on the numbers of trials included in the ERP averages showed no significant main effects or interactions for numbers of trials included as a function of group (all *p *> .13). Mean ± SD numbers of trials for the food pictures were 93.9 ± 23.7, 96.5 ± 22.1, and 99.0 ± 17.3 for the 10,000‐, 12,500‐, and 15,000‐step groups, respectively, and 91.5 ± 22.4, 96.7 ± 20.3, and 95.1 ± 22.1 for the flower pictures. Outcomes for the study were predetermined, and no changes were made prior to completing the study.

#### 2.4.3 Diet

Dietary intake was assessed by multiple uses of the Automated Self‐Administered 24‐hour Dietary Recall (ASA24) survey provided by the U.S. National Cancer Institute in conjunction with the National Institutes of Health (NCI and NIH). Using the web‐based ASA24, the participants were asked to record everything they ate and drank in the previous 24 h. The ASA24 includes a database of food items separated by food group. The participants were instructed by both audibly and by typed prompts in how to record meals and snacks. The serving size choices were supplemented with pictures. The system ensured that participants had included all foods consumed during the previous 24 h by using repeated prompts for “oft‐forgotten foods,” such as ketchup, soda, and butter. ASA24 has little error variance compared to interviewer‐based recalls and is more cost‐effective (Kirkpatrick et al., 2014; Subar et al., 2012). Participants completed ten 24‐h multiple‐pass dietary recalls throughout the duration of the study. Three recalls were performed randomly at baseline, and an additional recall was completed every 4 weeks during the intervention period (seven times total). The day of the week for this recall was chosen randomly.

### 2.5 Data analysis

We calculated the sample size a priori using the G*Power statistical software. The sample size was determined using a conservative effect size of .30, alpha of .05, and beta of .80. Based on this calculation, we needed 109 participants to complete the study. We estimated a 10% drop out rate and thus recruited 121 total participants for the study.

The statistical software PC‐SAS (version 9.3, SAS Institute, Inc., Cary, NC) was used to analyze the data. Alpha was set at *p* < .05. Means and SDs were calculated for all independent, dependent, and control variables. Effect sizes (Cohen's *d*) were calculated for the main effects for picture type and step condition. When calculating the effect size, the reference group was flowers for picture type and the 10,000‐step group for condition.

A 3‐Group (10,000; 12,500; 15,000 steps) × 2‐Picture Type (food vs. flower) mixed model repeated measures ANOVA was used to measure the effect of activity level on ERP amplitudes and subjective ratings in response to type of picture. Mixed model analysis was used to determine within‐ and between‐group interactions of activity level (steps/day) and neural indices of food motivation ERP amplitudes and latencies. A 3‐Group (10,000; 12,500; 15,000) × period (baseline vs. follow‐up) mixed model repeated measures ANOVA was used to evaluate the main and interactive effects for diet variables. The LSMeans procedure was used to evaluate significant main and interactive effects.

The influence of potential confounding variables on primary relationships was evaluated using partial correlation. Control variables for the ERP analysis included previous day steps, day of week, duration in study, and time of day. The control variables were analyzed one at a time and not all at once. None of the control variables had any influence on any outcome of interest. Because of this, reported *p* values in the results reflect uncontrolled values with degrees of freedom of two (numerator) and 76 (denominator) for the analysis of the main effect of step group and interactive effect of picture type and step group. For the analysis of the main effects of picture type the degrees of freedom were one (numerator) and 76 (denominator). All analyses were performed with participants in their originally assigned groups.

## 3 RESULTS

**FIGURE 2:**
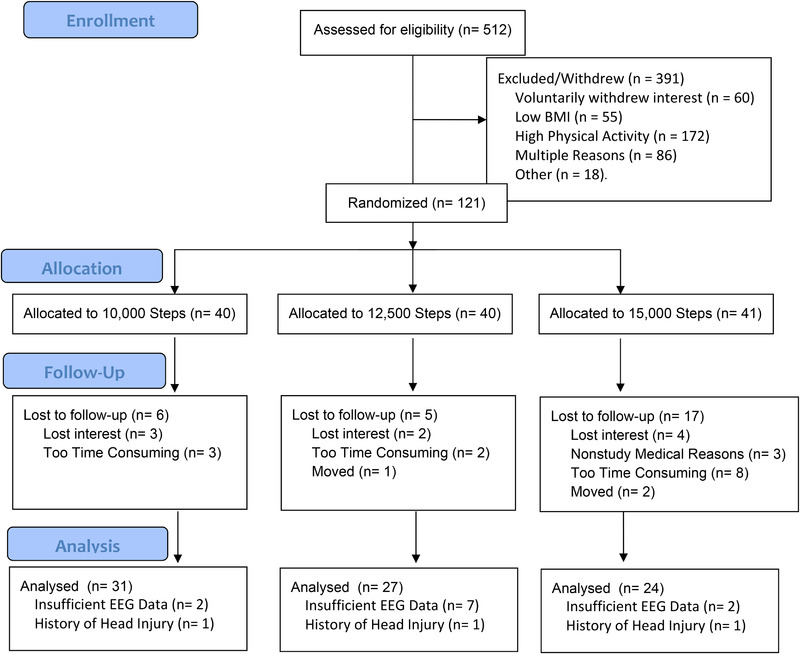
Participant flow diagram

The participant flow and inclusion diagram are presented in Figure 2. One hundred twenty‐one participants were initially randomized. Ninety‐three participants completed EEG testing, with 79 participants included in the final analysis. Demographics of the 79 participants included in the final analysis are reported in Table 1. Approximately 89% of participants were Caucasian, 8% Hispanic, and 3% Asian. Collectively, participants were studying 62 different majors. Drop out across groups was unequal; however, there was no difference in age, BMI, or baseline steps for women who completed the study compared to those who did not.

**TABLE 1:** Baseline demographics of participants who completed the study and were included in the data analysis

Variable	10,000‐step group	12,500‐step group	15,000‐step group	Total
	*n* = 31	*n* = 27	*n* = 21	*n* = 79
Age	18.5 ± 0.5	18.7 ± 0.4	18.4 ± 0.6	18.6 ± 0.5
Height (cm)	167.1 ± 4.6	165.2 ± 5.8	165.1 ± 7.0	165.9 ± 5.3
BMI (kg/m^2^)	22.9 ± 1.8	22.9 ± 2.4	22.8 ± 1.9	22.9 ± 2.0
Average steps at baseline	8032 ± 1587	8431 ± 1522	8392 ± 1384	8264 ± 1506

*Note*: Values are means ± SD.

Abbreviation: BMI, body mass index.

Mean step counts across the entire study are reported in Table 2. As expected, based on the intervention, there was a statistically significant difference between all groups in the correct direction for step count at study completion (*p* < .001). Adherence to the step recommendations was strong. Mean steps was 10,904 ± 927 (min: 7921.65, max: 13,266) for the 10,000 groups, 12,935 ± 1319 (min: 9664, max: 15,873) for the 12,500 group, and 14,077 ± 1276 (min: 11,808, max: 15,898) for the 15,000‐step group. Participants met the step recommendation 85% of the days in the 10,000‐step group, 77% of the days in the 12,500‐step group, and 70% of the days in the 15,000‐step group. Additionally, the difference in steps from baseline to study completion for all groups was significant (*p* < .001). Table 2 shows the change in steps and weight from baseline for each step group. There was no significant difference in weight change from baseline to follow‐up between any of the step groups (*p* = .839).

**TABLE 2:** Differentials for weight and steps across study

Variable	10,000‐step group	12,500‐step group	15,000‐step group		
	*n* = 31	*n* = 27	*n* = 21	*F*	*p*
Steps at baseline	8032 ± 1587	8431 ± 1522	8392 ± 1384	0.61	.549
Average steps over the course of the study	10904 ± 927^a^	12935 ± 1319^b^	14077 ± 1276^c^	45.59	<.001
Change in steps^*^	2912 ± 1842^a^	4504 ± 1625^b^	5685 ± 1780^c^	15.48	<.001
Change in aerobic steps^*^	1531 ± 1309^a^	2478 ± 1707^b^	3097 ± 1467^c^	7.57	<.001
Change in weight (kg)^*^	1.4 ± 2.6	1.8 ± 2.1	1.4 ± 2.1	0.18	.839

*Notes*: Values are means ± SD. *F* and *p* values represent differences in step groups at study completion. Means with different letters (a, b, c) are statistically different (*p* < .05).

*Difference refers to difference between baseline and study completion.

Valence and arousal were rated on nine‐point scales for valence (1 = extremely unpleasant, 9 = extremely pleasant) and arousal (1 = not at all arousing to 9 = extremely arousing or emotional). Collapsed across groups, flower pictures were rated as significantly more pleasant than food pictures (*t*[78] = 4.77, *p* < .001; mean valence rating for flowers = 5.98 ± 1.13, mean valence rating for food = 5.30 ± 1.19); there were no significant differences between participant arousal ratings for food and flower stimuli (*t*[78] = 0.90, *p* = .19); mean arousal rating for flowers = 4.40 ± 1.50, mean arousal rating for food = 4.56 ± 1.9). A 3‐Group (10,000; 12,500; 15,000 steps) × 2‐Picture Type (food vs. flower) mixed model repeated measures ANOVA for valence ratings showed no main effects or interactions as a function of step group (all *F*s < 1.57, all *p*s > .22). Similarly, there were no significant main effects or interactions as a function of step group for arousal ratings (all *F*s < 0.83, all *p*s > .44). Thus, although the flowers were rated as more pleasant than the food stimuli, the pattern of arousal and valence ratings did not differ by step group.

**FIGURE 3:**
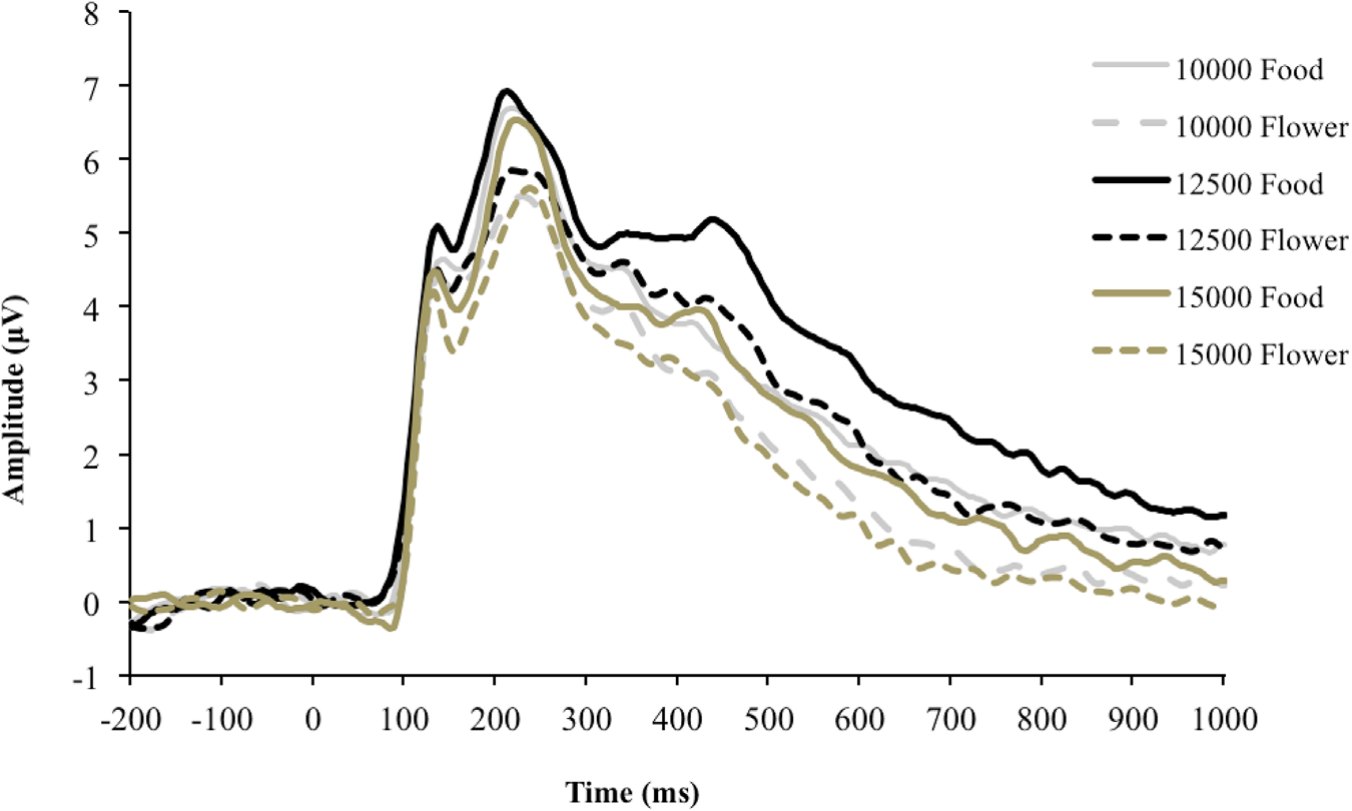
Event‐related potential waveforms: Food and flower values for each group averaged across sensors 65, 66, 70, 75 (Oz), 76, 83, 84, and 90. The P300 was measured as the average between 215 and 265 ms. The late positive potential (LPP) was measured as the average from 600 to 700 ms

**FIGURE 4:**
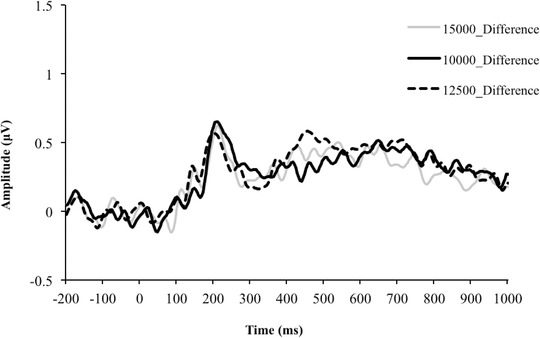
Difference waveforms: Food minus flower difference for each group averaged across sensors 65, 66, 70, 75 (Oz), 76, 83, 84, and 90. The P300 was measured as the average between 215 and 265 ms. The late positive potential (LPP) was measured as the average from 600 to 700 ms

P300 and LPP waveforms as a function of group and picture type are presented in Figure 3. Difference waves (food minus flower) are presented in Figure 4. Table 3 summarizes ERP amplitudes and latencies and the ANOVA results. There were significant main effects of picture type for the amplitude of the P300 and LPP, with food pictures having increased (i.e., more positive) amplitude compared to flower pictures (*p*s < .001). The effect size for P300 amplitude, P300 latency, and LPP amplitude using pictures of flowers as the reference was 0.28, −0.29, and 0.53, respectively. No interaction between step group and picture condition was found for the P300 amplitude, LPP amplitude, or P300 latency. There was, however, a significant main effect of group for the LPP amplitude. The 12,500‐step group had a significantly higher response in LPP amplitude to both food and flower pictures compared to the other groups (*p* < .004). The effect size for the LPP response using the 10,000‐step group as the reference was −0.60 when compared to the 12,500‐step group and 0.20 when compared to the 15,000‐step group. These results were not altered when controlling for age, baseline weight, weight change, day of the week, duration in the study, or the time of day the ERP assessment was conducted. There was no main effect of group for P300 amplitude or latency.

**TABLE 3:** Event‐related potentials by step group and picture condition

Variable	10,000‐step group	12,500‐step group	15,000‐step group	*F*	*p*
Picture Type	*n* = 31	*n* = 27	*n* = 21		
P300 amplitude (μV)				0.27	.766
Flower	5.3 ± 3.3	6.4 ± 3.6	5.4 ± 2.1		
Food	6.3 ± 4.0	7.2 ± 4.1	6.3 ± 2.2		
P300 latency (ms)				0.08	.926
Flower	235.5 ± 14.9	233.7 ± 16.8	235.8 ± 14.0		
Food	230.8 ± 13.2	230.5 ± 18.6	230.3 ± 9.9		
LPP amplitude (μV)				8.84	<.001
Flower	0.9 ± 1.1	1.7 ± 1.5	0.7 ± 0.9		
Food	1.8 ± 1.7	2.8 ± 1.9	1.5 ± 1.0		

*Notes*: Values are means ± SD. *F* and *p* values represent main effects between step groups for given ERP variable. There was no step‐group by picture condition interaction.

Abbreviation: LPP, late positive potential.

Table 4 describes the dietary data for participants who completed the study. The average total caloric intake at baseline was 2115 ± 527 kcal, and there was no difference between groups (*F*(2,120) = 0.59, *p* = .556). There was no main effect for period (*F*(1,74) = 1.10, *p* = .298) or step group (*F*(2,74) = 0.18, *p* = .835), but there was an interaction between period and step group for calories consumed (*F*(2,74) = 5.29, *p* = .007). A similar trend was observed for protein and carbohydrate consumption (see Table 4). Follow‐up analyses showed that the 12,500‐step group consumed fewer calories (*t*(1,74) = 3.35, *p* = .001, *d_z_* = 0.59), protein (*t*(1,74) = 3.24, *p* = .002, *d_z_* = 0.77), and carbohydrates (*t*(1,74) = 3.35, *p* = .001, *d_z_* = 0.51) at follow‐up compared to baseline (see Table 4).

**TABLE 4:** Baseline and follow‐up diet by step group

Variable	10,000‐step group (*n* = 34)				12,500‐step group (*n* = 34)				15,000‐step group (*n* = 24)					
	Baseline		Follow‐up		Baseline		Follow‐up		Baseline		Follow‐up			
	*M*	SD	*M*	SD	*M*	SD	*M*	SD	*M*	SD	*M*	SD	*F*	*p*
Energy (kcal)	2033	451	2069	396	2252	551	1964^a^	347	2063	772	2152	381	5.29	.001
Protein (g)	72	19	78	24	80	22	65^a^, ^b^	13	69	20	77	23	3.65	.002
Carbohydrate (g)	264	73	262	56	303^c^	92	261^a^	53	277	69	280	52	3.66	.030
Total fat (g)	80	22	81	17	84	26	77	16	80	75	85	21	1.82	.176

*Note*: *F* and *p* values refer to the period by step group interaction.

^a^ Baseline and follow‐up means are statistically different (*p*s < .05).

^b^ Follow‐up means are statistically different between the 10,000 and 12,500‐step groups (*p* = .02).

^c^ Baseline means are statistically different between 10,000‐ and 12,500‐step groups (*p* = .03).

## 4 DISCUSSION

The purpose of this study was to examine the effects of three progressively higher levels (dose‐response) of physical activity on ERP indices of attention allocated toward food pictures in freshmen college women. Findings from our study indicate that there was no preferential increase in ERP amplitudes or latencies to food over flowers due to increased habitual physical activity (i.e., due to step group). Notably, attention allocation (specifically LPP amplitude) to *both* food and flowers was influenced by physical activity level. However, the data presented in this study suggest that this relationship seems to be complex as the results were nonlinear. The nonlinear result should be interpreted with a degree of caution since we did not have a baseline assessment to verify equality of groups after randomization.

Findings from our study suggest an inverted U‐shaped relationship toward appetitive visual cues (food and flowers) with the group prescribed 12,500 steps per day demonstrating the highest LPP response. These results suggest that neural reflections to visual cues increase with increased habitual physical activity to a certain volume and that physical activity beyond this volume results in reduced neural reflections to visual cues. Therefore, the results from our study indicate that the influence of habitual physical activity on attention allocation to visual cues may be dose dependent and that the impact is not specific to food, but perhaps to pleasant stimuli such as food and flowers more broadly. This finding is consistent with previous research that shows the LPP is preferential to appetitive/pleasant stimuli (Gable & Harmon‐Jones, 2010); however, we did not include any negative stimuli in this study. Thus, it is possible that there is a different effect to negative or nonappetitive stimuli.

The current findings of an inverted‐U relationship with LPP amplitude are unique and can be interpreted in light of the few studies that have evaluated the neural reflections to food following changes in physical activity. Of the studies that have examined this relationship, the majority have been conducted following acute bouts of physical activity. Collectively, these studies have shown a decrease in neural reflections to visual food cues following acute exercise (Cornier et al., 2012; Evero et al., 2012; Hanlon et al., 2012). Although understanding the impact of acute bouts of exercise on neural reflections to visual cues is important in helping to understand the interplay between physical activity and attention toward food, these results do not take into account the body and brain's ability to adapt. The mechanisms that drive these changes represent acute physiological changes rather than chronic adaptation.

To our knowledge, there are few studies that have attempted to measure neural reflections to food following a prolonged period of increased physical activity (Cornier et al., 2012). In this study, the authors used a pre‐experimental pre‐ and posttest design and measured change in neural response (evaluated using fMRI) following a progressive 6‐month treadmill intervention in 12 participants (five women, seven men, mean BMI 33.3 ± 4.3 kg/m^2^, and mean age 38.2 ± 9.5 years). The exercise load targeted 500 kcal increase per day (an additional 2500 kcal/week). The results of this study demonstrated that the neural response to food cues decreased compared to nonfood cues, particularly in the bilateral insular cortices, which are known to be important in regulation of food intake. While the design of our study was different, the results of both studies taken together support the possibility that chronic physical activity can influence the neural reflections to food cues. However, our study indicates that this relationship is not linear and more quadratic, and that the neural response is not food‐specific.

The results from our study may indicate the existence of a physical activity threshold. According to the inverted‐U hypothesis concerning physical activity and cognitive performance, it is possible that there may be an optimal physical activity workload at which neural activity is the highest and cognitive function is most effective (Kashihara et al., 2009). However, Once this point is exceeded, cognitive performance decreases, creating an inverted‐U result. This inverted‐U relationship has been observed in a number of studies that have evaluated the impact of acute exercise on cognitive function (Bender & McGlynn, 1976; Chmura et al., 1994; Kashihara et al., 2009; Levitt & Gutin, 1971; Reilly & Smith, 1986; Salmela & Ndoye, 1986; Sjoberg, 1975). However, our study is the first, to our knowledge, to observe the existence of this type of relationship with habitual physical activity. Based on our findings it is possible that 12,500 steps per day is the optimal point for heightened attention and neural reflections to visual cues. Exceeding this volume of physical activity may result in a decrease in neural reflections toward all appetitive cues. While this finding is provocative, replication is needed to confirm and better describe this relationship.

The participants diet was also measured to examine how the observed neural changes were potentially related to diet behavior. The results of the study suggest that energy intake was significantly lower from baseline in the 12,500 group and the rate of changes was significantly different in this group compared to the 10,000‐ and 15,000‐step groups. Interpreting this data is a little challenging since the LPP was higher in the 12,500 group, suggesting that motivation to consume food might be elevated. However, it is possible that the reduction in food consumption had an impact on neural reflections on food, and the lower energy intake resulted in greater LPP amplitude. More research is needed to clarify this relationship.

One interesting and difficult aspect of the current findings is disentangling the LPP and P300 results. There were no significant main effects or interactions for the P300, while the LPP showed increased amplitude in the 12,500‐step group regardless of food or flower presentation. The P300 is thought to represent a rapid allocation of attention to motivationally relevant stimuli with utility for stimulus categorization and decision‐making, while the LPP is thought to be a more sustained engagement and processing of the emotionally relevant information based on the significance of the stimulus to the participant (see Schupp et al., 2007; Hajcak & Foti, 2020). Several researchers have questioned whether the P300 is simply a rapid and transient LPP (e.g., Hajcak & Foti, 2020); however, seminal early work suggests that the primary difference between the P300 and LPP is the P300 reflecting the speed of a decision to categorize and respond to the stimuli and the LPP reflecting continued sustained processing after stimulus categorization (Donchin, 1981; Verleger, 1997). Thus, current differences may reflect continued processing that is enhanced with a moderate habitual exercise load but does not influence initial decision‐making. Future studies are needed with different speeds of stimulus presentation in conjunction with exercise to ascertain this possibility.

There are a number of studies that have examined dietary intake following exercise interventions of varying intensities and durations (Caudwell, Finlayson, et al., 2013; Caudwell, Gibbons, et al., 2013; Donnelly, Hill, et al., 2003; Rocha et al., 2016). The majority of studies that have examined changes in activity and diet have not observed any change in energy or macronutrient consumption compared to controls (Caudwell, Finlayson, et al., 2013; Caudwell, Gibbons, et al., 2013; Donnelly et al., 2014; Donnelly, Kirk, et al., 2003; Rocha et al., 2016). However, there are a few studies that have shown reduced energy intake with prescribed exercise over an extended period of time (Bales et al., 2012; Foster‐Schubert et al., 2012; Kirkwood et al., 2007; Nieman et al., 1990). These findings tend to support the results observed in our study. There was no change in both the 10,000 and 15,000 steps groups over the course of the study, but energy intake was reduced in the 12,500‐step group. It is possible that the volume of habitual physical activity that a person participates in has an impact on their response to dietary decision and subsequent energy intake. These results are supported by the energy flux hypothesis, which proposes a nonlinear (inverted‐U) relationship between habitual physical activity and energy intake (Foright et al., 2018).

As with any experimental study, there are some limitations that should be discussed. First, while all step groups experienced some drop out, there was slightly higher than expected drop out in the 15,000‐step group. This level of attrition is not uncommon in exercise studies where a high volume of activity is prescribed for a long period of time (Donnelly, Hill, et al., 2003). In addition, because the study design did not include a pretest, we were not able to evaluate pre‐ to postintervention change in dependent variables. The lack of a pretest also did not allow us to verify groups were equal at baseline on dependent variables. However, the groups were randomly assigned at baseline allowing us to work under the assumption of general equality between groups (Marczyk et al., 2005). The lack of any observed difference for other variables including BMI, body composition, steps per day, and caloric consumption supports this assumption of general equality at baseline. However, our results should be interpreted with this limitation in mind, and future studies are needed to verify the results.

Despite these limitations, our study makes some significant additions to the current literature. First, the study is the only one to use an experimental design to answer the question of a relationship between habitual physical activity and neural reflections to food cues. We not only measured this relationship but also used different activity levels to do so. This allowed us to see how neural reflections vary with differing levels of physical activity. It further enabled us to identify a possible threshold, beyond which further physical activity has a dampening effect on neural response. Additionally, the current study is the largest study to date to measure neural reflections to food cues following chronic physical activity. Because the study was adequately powered, we were better able to evaluate the proposed relationships. This is also the only study that has attempted to change physical activity patterns and evaluate how it influences attention allocation to food pictures using ERPs. Using ERPs as the method of measurement complements and strengthens results found in fMRI studies, as ERPs have greater temporal resolution indicating the timing of neural reflections to visual cues. Finally, the addition of the behavior measure of food consumption adds additional insight into the relationship between changes in neural responses to food and dietary behavior.

## 5 CONCLUSION

Based on the findings from this study and other studies, it appears that both acute and chronic physical activity have the ability to alter neural reflections toward food cues. However, our study indicates that the chronic change in neural reflections is not specific to food. Thus, it is possible that there is a more global neural response to appetitive cues in general since both food and flowers are pleasant stimuli. In addition, the chronic neural response to food and flowers as a result of prescribed lifestyle physical activity may not be linear and seems to demonstrate an inverted U‐shaped relationship. What this means for sensitivity to food cues, and implications for energy intake, are not clear since the neural response was not food‐specific. However, we did see a change in energy intake in the same group (12,500 steps) that observed changes in LPP response, suggesting that the neural changes to food are related to the behavioral changes to food. Future research should evaluate these findings in other populations since these findings are limited to first year female college students. In addition, studies that evaluate more specific training protocols of various intensities are needed to better describe how intensity of activity might influence these relationships.

## CONFLICT OF INTEREST

The authors declare no conflict of interest.

## AUTHOR CONTRIBUTIONS

Sharla E. Compton is the first author of this paper and was involved in all aspects of the study. She came up with the research question, drafted the proposal, directed data collection, assisted in data analysis and was the main person who drafted the manuscript. She has critically reviewed this paper, approved its submission and agreed to be accountable for the accuracy and integrity of the information.

Bruce W. Bailey is the senior author of this paper and has been involved in all aspects of the study. He has approved the final version of this paper and approves the submission. He agrees to be accountable for the integrity of the study.

Michael J. Larson, James D. LeCheminant, and Larry A. Tucker1 are all coauthors and have made substantial contributions to the conception and design of the work. They have reviewed the manuscript critically for important intellectual content. They give their approval for the paper to be published. They have also agreed to be accountable for the paper and will answer any question related to the accuracy or integrity of the manuscript. ML was in charge of the EEG measurement and analysis.

### PEER REVIEW

The peer review history for this article is available at https://publons.com/publon/10.1002/brb3.2590.

## Data Availability

The data that support the findings of this study are available from the corresponding author upon reasonable request.
